# Changes in the representation of space and time while listening to music

**DOI:** 10.3389/fpsyg.2013.00508

**Published:** 2013-08-06

**Authors:** Thomas Schäfer, Jörg Fachner, Mario Smukalla

**Affiliations:** ^1^Department of Psychology, Chemnitz University of TechnologyChemnitz, Germany; ^2^Department of Music and Performing Arts, Anglia Ruskin UniversityCambridge, UK

**Keywords:** space, time, music, altered states of consciousness, absorption, embodiment

## Abstract

Music is known to alter people's ordinary experience of space and time. Not only does this challenge the concept of invariant space and time tacitly assumed in psychology but it may also help us understand how music works and how music can be understood as an embodied experience. Yet research about these alterations is in its infancy. This review is intended to delineate a future research agenda. We review experimental evidence and subjective reports of the influence of music on the representation of space and time and present prominent approaches to explaining these effects. We discuss the role of absorption and altered states of consciousness and their associated changes in attention and neurophysiological processes, as well as prominent models of human time processing and time experience. After integrating the reviewed research, we conclude that research on the influence of music on the representation of space and time is still quite inconclusive but that integrating the different approaches could lead to a better understanding of the observed effects. We also provide a working model that integrates a large part of the evidence and theories. Several suggestions for further research in both music psychology and cognitive psychology are outlined.

Time and space—time to be alone, space to move about—these may well become the great scarcities of tomorrow.

Edwin Way Teale

## Introduction

The way people orient themselves and behave in the world essentially depends on spatial and temporal magnitudes. Simple actions require a coherent and appropriate representation of the surrounding space as well as of the chronological time that is intrinsic to physical and biological processes. People may initially have the naïve impression that (1) ordinary (Euclidean) space and chronological time are invariant physical magnitudes, and that (2) orientation and behavior work best when people are able to represent both space and time as accurately as possible. Both assumptions have been proven wrong, however. From a physical standpoint, modern physics—going back to Einstein's work in the early twentieth century—and especially quantum mechanics have abandoned the idea of invariant space and time. Instead, it is known that space can be bent or dilated and time can pass at various speeds, depending on the perspective of the beholder. Of course, these physical peculiarities are far from our human scope of experience. But also from a psychological point of view, it appears that our representation of space and time is alterable and malleable. Both entities are a product of conscious and unconscious processing, affected by perceptions, memories, moods and emotions, our interests and current goals, and our physiological condition. Neither space nor time is a characteristic of objects or events in the outside world that we “perceive.” Rather, both are characteristics of our mental representations of what we categorize as objects or events.

Psychology has only just begun to acknowledge the changeableness and dynamics of people's representations of space and time (see Clark, 1998; Michon, 1998; Buhusi and Meck, 2005; Vaitl et al., 2005; Woods et al., 2012). Because people describe the world in terms of space and time continually, it should be of the utmost interest to psychologists to consider the representation of these concepts when thinking about psychological processes in each domain of their research. To date, this has not been the case; when space and time appear in psychological processes they are consistently thought of as static dimensions that can be measured by rulers and clocks. But because space and time are representations of subjective mental operations—and therefore flexible and malleable—such an approach may contain considerable pitfalls.

The importance of spatial and temporal information to the understanding of psychological processes becomes particularly evident when these processes are considered from the perspective of embodied cognition. According to the *embodied cognition* (or *embodied mind*) hypothesis, cognitions and mental operations are influenced and shaped by the state and condition of the body and its spatial and temporal positioning. That is, thoughts, concepts, categories, plans, mental images, and intentions cannot be considered isolated entities but are all contingent on the body. The rationale for this hypothesis is that cognitions and mental operations are always related to intentionality, that is, to the question of *what can be done* with them (see, e.g., Wilson, 2001; Gallagher, 2013). The body's positioning refers to perception, proprioception, and motor action, which are all characterized by the integration and fine tuning of spatial and temporal information.

We sought to analyze the alteration of the subjective representation of space and time using a simple and ubiquitous everyday behavior that is known to affect people's normal state of consciousness: listening to music. Music, as (1) a continuous flow of auditory input and (2) something that is done or performed by someone, evidently has the potential to affect the mental representation of space and time. We give an overview of the research that has been done with music and that has provided evidence of the multiple ways in which listening to music can influence the ordinary representation of space and time. We also discuss models and theories that have been adopted to explain these influences, including psychological, neurobiological, and phenomenological approaches. Finally, we ask how the results of this review can help us better understand the topic of music and the embodied mind and try to delineate suggestions for further research in both music cognition and cognitive psychology.

## Music and the representation of time

There is both experimental evidence and evidence from subjective reports of altered representation of time while listening to music. Experimental research has been concerned with time estimation, that is, with *judgments of the duration* of certain intervals and—more rarely—with the *subjective flow of time* during these intervals. In addition, there are studies that analyzed reports about *subjective experiences* collected in surveys and interviews. We summarize both lines of research along with the most prominent approaches to explaining their findings.

### Experimental evidence of altered representation of time

Human time estimation depends on what stimuli are present and what is happening in the interval to be estimated. To illustrate processes of overestimation and underestimation of time intervals more precisely, researchers speak of a “leftward shift” (←), meaning the estimated duration of events is shorter than the chronological extent measured (as reference), or a “rightward shift” (→), meaning estimated durations are longer (see Meck, 1996, 2005).

Typically, music has been thought to *shorten the estimation of interval length*. Rai (1973) found that music-filled intervals were estimated as shorter (←) than noise-filled, light-filled, and unfilled intervals, respectively. In line with Rai's results, findings from a series of studies using simple waiting situations showed that time intervals were estimated as being shorter (←) when music was played (MacNay, 1995; North and Hargreaves, 1999; Roper and Manela, 2000; Guéguen and Jacob, 2002; Kämpfe, 2011), and Droit-Volet et al. (2010) recently demonstrated a decrease (←) in people's time interval estimates while listening to music using a temporal bisection task.

Arousal, familiarity, affective response, and music preference have been considered explanatory variables in the relationship between music and time estimation. When investigating estimates of advertisement durations, Kellaris and Mantel (1996) found an underestimation (←) of the duration of music-filled intervals, which was even stronger when the music was *more arousing* than *less arousing*. In contrast, other researchers have found that loud music (which is more arousing than soft music) led to longer estimates (→; Kellaris and Altsech, 1992), as did faster music (which is more arousing than slower music;→; Oakes, 2003; Droit-Volet et al., 2013).

Bailey and Areni (2006) investigated the role of *familiarity* and found that the decrease of time interval estimates (←) was more pronounced when the music under study was familiar as opposed to unfamiliar. Similarly, Yalch and Spangenberg (1990) found that shoppers in a department store reported shorter time estimates (←) when music was played that they would usually listen to—relative to music they would not usually select. In contrast, studies by Gulas and Schewe (1994) and Yalch and Spangenberg (2000) yielded time duration estimates that were longer (→) under the influence of familiar music than under the influence of unfamiliar music.

Given that previous research showed that *affective responses* to music might influence time perception, as well (e.g., Kellaris and Kent, 1992), the effect of familiarity may have been mediated by affective responses. However, Bailey and Areni (2006) ruled out this explanation in their study; and Droit-Volet et al. (2010) demonstrated that music in major mode (which is more strongly associated with positive affect) and music in minor mode (which is more strongly associated with negative affect) do not differ in their influence on time perception.

Not least, it has been suggested that *music preference* mediates music's influence on time perception. Lopez and Malhotra (1991) found that interval durations were overestimated (→) when people listened to their least preferred music and underestimated (←) when they listened to their most preferred music. Hui et al. (1997) found that positively valenced music increased perceived wait duration (→) and Cassidy and MacDonald (2010) found that exposure to self-selected music resulted in overestimation of time (→) as compared to experimenter-selected music, but Cameron et al. (2003) again found that listeners evaluated waiting time durations as being shorter (←) the more they liked the music that was played in the background.

By way of summary, music listening typically leads to a leftward shift, that is, an underestimation of time intervals. Taking familiarity, arousal potential, and likeability into account, however, leaves us with a rather puzzling picture: In some cases, time interval estimates are in a positive relationship with these variables but in other cases, the relationship is negative.

### Evidence from subjective reports of altered representation of time

Apart from the results of experimental studies on time estimation, there is also evidence from subjective reports collected in surveys and interview studies. The vast majority of such reports come from research about music listening and altered states of consciousness (ASCs), because an altered representation of time is one of the predominant characteristics of ASCs (Dietrich, 1996; Vaitl et al., 2005; see also Studerus et al., 2010). As will be apparent below, the relationship between these studies is rather loose. Most of the studies had a focus other than time experience in music listening, so that they came across changes in the representation of time rather incidentally.

The most widely reported experiences with regard to music listening are the feelings of *timelessness* and *time dilation*. Hills and Argyle (1998) found that musical experiences are highly similar to religious experiences in that both are characterized by the feeling of timelessness. Kohlmetz et al. (2003) investigated the performance of a pianist playing for 28 h and found him to reach meditative states characterized by “the loss of the external framework (time, space, and bodily sensation) and mental content (inner and outer perception)” (p. 174). In her profound qualitative investigation of everyday music listening, Herbert (2011, p. 76) summarized that “typically, time may appear to feel protracted in the present moment of experience, but may seem to have passed quickly when that experience is remembered.”

Hutson (2000) described the ritual character of rave parties where people can experience ASCs that include the feeling of timelessness. Evidently, such experiences are an effect not only of listening to music but also—and probably even more so—of dancing to the music. Dancing translates music to a bodily expression and thereby is an example par excellence of the embodied repercussion of outer stimuli (sounds) on the inner experience. Remarkably, the occurrence of such extraordinary states does not depend on psychoactive drug use (see also Baldemair, 2003). However, triggering and binding to corresponding endogenous neurotransmitters and their receptor systems in the brain mediate the pharmacokinetics and dynamics of induced psychoactive drug action (Julien et al., 2008). Thus, induced drug action on music experience represents a model of endogenous neurotransmitter action on music processing (Fachner, 2000). Fachner (2000, 2008) showed that cannabis can intensify musicians' time perception so that they experience time as dilated, which provides them greater temporal scope to develop and perceive musical structures and elements. He argued that cannabis affects the perception of time, space, and frequency, which intensifies the perception of temporal–spatial sound figures and leads to psychoacoustic quality intensification.

In a large-scale qualitative study, Gabrielsson and Lindström Wik (2003; see also Gabrielsson, 2001, 2002, 2011) found that strong experiences with music were often characterized by changes in time perception (time disappears, goes slower or faster). Exemplary statements of interviewees were “We stood outside time,” “Time had stopped; it felt as a single long moment,” “I often have a feeling of eternity in connection with the experience of music” (Gabrielsson and Lindström Wik, 2003, p. 174). In addition, altered perception of time was often related to feelings of trance, ecstasy, or transcendence. Not least, an interesting description of an alternative conceptualization of time within music was given by Roswell (1986), who detailed that Indian music operates without concepts such as counting and rhythm and where time is rather latent. About temporality in Indian music he stated that “*structure is mutable and substance is malleable*” and that “forms are fluid and constantly shifting” (p. 233).

In sum, studies about the subjective experience of time under the influence of music in intense listening situations typically found time being experienced as either slowing down or disappearing. However, the evidence of subjective experiences either relies on only a few participants or refers to rather broad categories (such as changed perception of time during intense experiences with music). Thus, evidence of such subjective experience of time is accordingly scarce and conclusions are limited.

### Approaches to explaining altered representation of time under the influence of music

We now introduce several theoretical approaches to explaining the effects of music on the representation of time. We discuss attention-based models, memory-based models, phenomenological models, models that refer to absorption and ASCs, and some basic neurophysiological explanations.

#### Attention-based models

The most prominent approaches to explaining the distortions of time interval estimates under the influence of music are attention-based models and memory-based models of human time processing (e.g., Block and Zakay, 1996; Brown, 1997; Zakay and Block, 1997). For instance, according to the *attentional gate model* (Zakay and Block, 1997), music distracts cognitive processing away from time processing, which leads to a decrease in the estimation of elapsed time in retrospect and to the feeling that “time flies” in the immediacy of the current experience.

Our sense of subjective time is thought to fluctuate in relation to clock time according to the amount of information we receive per second. Tse proposed the idea of a simple counter model, arguing that the brain “has access to the approximate constant rate of its own information processing” (in Eagleman et al., 2005, p. 10369). For example, if one bit of information processed is interpreted as one unit of objective time, then—in moments when attention is shifted from and/or increased on (a series of) engaging percepts—two or three bits of information would be counted again as one unit of objective time, “creating the illusion that time and motion had slowed down” (Eagleman et al., 2005, p. 10369). Only a specific, individually and situationally relevant excerpt of sensory data is accessible to our consciousness (with an upper limit of around 15–20 bits/s), as outlined in an information-based model of the optimization and throttling of sensory input and output during top-down and bottom-up processing of sensory information (Spreng and Keidel, 1963). However, to date, the problem of how bits of information can be counted in musical selections remains unsolved:

It depends on the individual, how well he knows the given musical style, his ability to codify musical events, and his ability to concentrate during the performance …. Experience and training thus have a direct relation to the amount of “information” that can be grasped from a musical phrase. (Mountain, 1989, p. 4).

Nonetheless, is has also been argued that the inner pacemaker is influenced by physiological arousal (Zakay and Block, 1997). If music increases arousal, the pacemaker is thought to accelerate, resulting in an overestimation of the elapsed time in retrospect (see Oakes, 2003; Droit-Volet et al., 2013). As we mentioned above, however, some studies found just the opposite: Higher arousal resulted in shorter estimates (Kellaris and Mantel, 1996). The authors argued that “the presence of arousing music may have occupied attentional resources that would otherwise have been devoted to processing the information in the test ads, with attendant effects on memory-based temporal inferences” (p. 510). That is, the influence of arousal on time estimation may be mediated by attention. Moreover, arousal has also been shown to influence music preference (Schäfer and Sedlmeier, 2011), so that higher arousal may lead to a higher preference and, in turn, to shorter time interval estimates. And, not least, the focus of attention—being either broad or narrow—depends on affective states (Friedman and Förster, 2010) and motivational intensity (Harmon-Jones et al., 2011). Effects of affect and motivation on time estimation could therefore be explained by the mediating effect of attention. This has not yet been investigated with regard to music listening.

In sum, attention-based approaches in their current form can explain some—but by no means all—of the findings about the influence of music on the estimation of time intervals. A major problem in models such as the attentional gate model is that they have two degrees of freedom: the pace of the pacemaker and the degree of attention directed to the time. Both these variables can be influenced by musical characteristics such as tempo, loudness, emotional expression, complexity, familiarity, or likeability in manifold ways. The models are still not precise enough to allow clear predictions about which of the two parameters is influenced by which of the musical characteristics so that eventually they sometimes appear to explain any empirical result, regardless of in which direction it goes.

#### Memory-based models

Memory-based models of psychological timing (see Ornstein, 1969; Kellaris and Mantel, 1996) reason that the more information is processed in a period of time, the more “traces” remain in memory, which results in longer time estimates in retrospect. Music—as compared to silence—would require more processing and produce more traces in memory.

Whether traces are built in memory and whether they can be retrieved easily later depends on several variables, the most important of which are engagement in the listening experience and evoked affect. Pöppel (2000)—in line with James (1890)—used the term *time paradox* to describe when time periods with a dense event structure that are subsequently recalled are estimated as prolonged when the events are engaging, even when the physical duration is objectively short. It seems that “time judgments can distort, recalibrate, reverse, or have a range of resolutions depending on the stimulus and on the state of the viewer” (Eagleman et al., 2005, p. 10370; see also Kramer, 1988). The “state of the viewer” seems to depend especially on the affective state. Frightening film passages were recalled as being longer than sad or so-called neutral excerpts (Droit-Volet et al., 2011). State-dependent cognition and state-dependent recall of time passages and cross-temporal binding of events are more profound when they are connected to strong emotional and personally meaningful experiences (Fachner, 2010, 2011).

In sum, memory-based models can account for the observation that people overestimate temporal durations in retrospect under the influence of music. The models also claim that this effect is more pronounced when listeners are more engaged in the experience or encounter stronger affect. There are, however, only a few studies that empirically tested these conjectures. In addition, research has been done to compare memory-based models with attention-based models, with results usually favoring the attention-based explanations (see North and Hargreaves, 2008).

#### Phenomenological models

Some scholars have suggested that what we call time “perception” might be better understood as a mental construction that is continuously updated during “ordinary” mental functioning (see Fraisse, 1963, 1984; Thomas and Brown, 1974; Block and Reed, 1978; Poynter, 1983; Le Poidevin, 2009). This construction may be built through single events in human consciousness (*temporal markers*, such as the number of breaths, the number of lines of a paper read) and the fact that people have learned to label a specific number of such events with a specific declaration of how much time they lasted (see Flaherty's, 1999, concept of synchronicity below; see Betsch et al., 2010, for the influence of stimulus frequency on duration judgments). In this respect, the experience of time is always a *relative* experience (Michon, 1998). Under the influence of music, this construction may be changed more or less drastically in that there are no more single events in the consciousness but only the music that expands to a boundless moment. Next we present two influential phenomenological models. The first is centered around the subjective flow of time; the second is Flaherty's (1999) philosophical–sociological model.

The notion that time is a construction rather than a perception is intrinsic to phenomenological approaches to time consciousness. With reference to Kant, Husserl, Heidegger, Merleau-Ponty, James, and others, scholars have delineated a model of the phenomenology of temporality, the flow of time, and the experience of what we call the *present* or the *now* (e.g., Varela, 1999a,b; Gallagher and Varela, 2003; Pockett, 2003; Gallagher, 2009, 2011, 2013). Central to the model is the notion that the relationship between the experience of time and the temporal structure of events in the real physical world is neither linear nor following a fixed rhythm or pulse. What we experience as now or present is a mental act of integrating a certain number of perceivable stimuli (represented by single post-synaptic potentials) into a single unit of content within consciousness (referred to as a *time object*). Time objects are embodied entities, as their function is to nurture cognitions that are directed toward potential intentional actions of the individual. They seem to be established by temporary synchronization of cell assemblies across the brain and last between 0.5 and 3 s (see Pöppel, 1988; Linke, 2005). These units can be either strung together without a gap or separated by gaps of varying duration. The gaps facilitate the involvement of the ego, which integrates the single contents of consciousness into the absolute flow of a person's coherent past. Without these gaps there is no ego and we would live in an everlasting present. This eventually means that we have an experience of the flow of time only because we have conscious access to our memory. (As Julian Barnes puts it in his lovely novel *The Sense of an Ending*, “this personal time, which is the true time, is measured in your relationship to memory.”)

The phenomenological model of temporality further states that the number and order of perceivable stimuli that get bound together to form a unit is flexible, as is the temporal rhythm of succeeding units. This simply means that the experience of temporality is malleable: What we perceive as now or present can have a very different duration in physical time. Hence, the “flow of time” can change because the succession of experienced moments of “nowness” occurs against the background of memory—a notion quite similar to Flaherty's (1999) approach discussed below. The length and temporal succession of the single units depend on memory, personality, affect, intentions, physiological conditions, and of course currently perceivable stimuli. Music may influence both the length of the units (how many notes or musical elements are bound together) and the existence and duration of gaps between them. Music that is more captivating will cause the listener to become addicted to the flux of pure perception, giving rise to only a few moments in which the ego can make a conscious judgment of elapsed time. As a result, time might seem to disappear (when there are no gaps at all), to speed up (when temporal judgments are made rarely), or to slow down (when the music is boring or disagreeable so that temporal judgments are made more often than usual).

Flaherty (1999) presented a philosophical–sociological approach to human time processing, starting with the notion that time is a construct of consciousness and in that way affected by societal conventions and the inner processes of the individual. He proposed three forms of temporal experience: “Protracted duration” refers to the immediacy of the current experience and to the feeling that time is passing more slowly than usual. This happens in the course of very intense, exceptional, or non-routine experiences, as they are characterized by a denser structure of to-be-processed information. Arstila (2012), for instance, analyzed the experience of time slowing down in high-anxiety situations such as accidents. He argued that this experience is due to not only an acceleration of cognitive processes but also the awareness of the fact that things are taking *less time than usual*. This is where memory and embodiment come into play. What is retrieved from memory and compared to the current situation is the embodied knowledge of the specific amount of time things usually take (e.g., how long it takes to read a word, to speak a sentence, or to walk a distance of 100 m). The second and most prevalent form of temporal experience is “synchronicity,” which means that subjective time is constantly synchronized with conventional clock time, enabling the coordination of internal and social processes. Synchronicity is the usual way we experience time, during which people do not have any extraordinary experiences such as a speeding up, a slowing down, or even a disappearance of time (see also Hammond, 2012). Finally, “temporal compression” refers to the reconstruction of temporal experience retrospectively. Flaherty proposed two possible means of temporal compression that can be directly related to music listening. When music is listened to more casually, the listener's mind is thought to function in a rather automatic way, so that “time will be experienced *and* retroactively constructed as having flowed quickly” (Holmer Nadesan, 2002, p. 259). On the other hand, when listeners are captivated or absorbed by music or have very intense or exceptional experiences (see protracted duration) “time will be experienced in the present as passing slowly but may be *retroactively* interpreted as having passed quickly” (Holmer Nadesan, 2002, p. 259). In addition, this effect can change when the experiences made are strong enough to leave traces in episodic memory, which would lead to the experience being retroactively interpreted as having lasted longer (see also Croom, 2012; Schäfer et al., 2013; for an overview about music and engagement).

Flaherty's (1999) model can explain the prospective experiences of time slowing down (when the music is captivating) and time speeding up (when the music is only in the background) as well as the retrospective experiences of time having passed slowly (when the listening experience was very significant) and time having passed quickly (when the listening experience was captivating but not very significant). Apparently, the model adopts the idea of memory storage mentioned above and is a good candidate for explaining most of the results found in the time interval estimation studies. In particular, it may explain why music-filled intervals are remembered as having lasted longer only when the experience was outstanding or personally involving. And of course, this is an interesting point, given that most of the studies mentioned above used very short intervals and were conducted in artificial lab settings. That is, whether the passage of time while listening to music is experienced as being more quickly or more slowly depends on the emotional involvement of the listener and on the effects the music causes in that listener.

#### Models that refer to absorption and ASCs

Another line of explanation for the influence of music on timing has focused explicitly on the role of absorption and ASCs in music listening. Most studies about ASCs have been based on drug research and emphasize the role of various neurotransmitter processes, such as serotonergic (Wittmann et al., 2007a,b; Sysoeva et al., 2010), cannabinoid (Mathew et al., 2002; Fachner, 2009), dopaminergic, and cholinergic (Meck, 1996; Rammsayer, 1999) interactions with altered time perception and (re)production (see also Shanon, 2001). Studies of patient populations and drugs indicate variations in the scaling of musical events attributable to the deceleration and acceleration of the internal clock and the internal representation of perceived elements when reproducing or estimating time intervals in the millisecond-to-second and the second-to-minute ranges (Meck, 2005). Thus, it is reasonable to expect endogenous neurochemistry to be activated in various ways when experiencing ASC phenomena and music.

Research on ASCs has shown that music is one of their most important triggers. A constant musical rhythm together with monotonous and repetitive elements seems to lead the listener to states of absorption, trance, or ecstasy, where the focus of attention turns to an inner view. That is, the representation of objects and events in the outside world diminishes somewhat and attention is focused instead on memories and emotions elicited by the music. Time just ceases to be important and disappears when attention is captivated by memories and emotions (Fachner, 2011). This need not mean, however, that listeners—when asked to state how long a musical experience of a certain duration has been—are not able to give a very accurate judgment. At the moment they are asked, they might well-remember how many songs they had listened to and how long these songs generally last, so that they are able to reconstruct the elapsed time in retrospect.

When reviewing the psychobiology of ASCs, Vaitl et al. (2005) argued that drumming and dancing have been used since time immemorial to induce trance, which includes a distortion of time representation. The distortion seems to depend on the rhythm as an external timekeeper inducing entrainment of internal biological processes. Discussed effects of such auditory-driven entrainment in trance rituals are concomitant changes of brain activity, as observed in the theta range (Neher, 1961; Berger and Turow, 2011). However, in earlier research, Rouget (1985) stressed that a successful trance induction depends on the social setting, personality characteristics, and rhythmic body movements, which are all variables pertaining to embodiment. His descriptions of music-induced ASCs—namely, “ecstasy” and “trance”—are connected to the amount of body movement within contexts aiming to induce an ASC. Although trance is connected to dance, ecstasy describes an inwardly turned ASC accompanied by music (Fachner, 2011).

As the alert reader has certainly noticed, the approaches regarding absorption and ASCs refer to the central role of attention, as well. That is, absorption and ASCs do not provide an alternative explanation for the effects of music on psychological timing but they can provide an explanation for how these effects are mediated by additional variables such as the music's emotional expression, its likability, and the personality and the state of the listener.

It is an open and interesting question whether music always elicits ASCs to some degree. Of course, an appropriate answer to this question depends on the definition of an ASC. Following Vaitl et al. (2005), who suggested that we can experience various states of consciousness even when we are awake, we agree that music—that we do not dislike and that does not distract us from other activities we are engaged in—induces a state of “trancing” (Becker, 2004), a transient state of being dissociated from everyday life, a feeling of being temporarily “out of time” (see also Herbert, 2011). The role of music in such experiences may be that it acts as a trigger that stirs memories and emotions, enticing the brain to create manifold associations, resulting in an “inner view.” Such increased imaginableness may be visible in altered brain activity, on which future studies may shed some light. Moreover, it remains to be investigated how musical characteristics interact with the characteristics of the listener when an ASC develops. Music can be fast, loud, and with a steady beat, as, for example, in gospel music, leading to religious rapture; or the music can be slow, solemn, and spherical to accompany contemplative worshiping (Söhngen, 1967). For Rouget (1985), music creates emotional conditions, and it structures the chronological order of symbolic events, especially in ceremonial settings in which it is intended to alter consciousness states for individual or group ritual purposes.

#### Neurophysiological models

The effects of music on the representation of time can be underpinned by neurobiological evidence. We first discuss approaches related to potential neuronal correlates of an inner clock and then present evidence specifically related to absorption and ASCs.

As we have shown above, some models on interval timing incorporate the idea of a pacemaker or internal clock that is task dependent and state dependent (see Fachner, 2010). Regularly emitted pacemaker pulses are temporarily stored in an accumulator, and a task-related number of criterion pulses are stored in reference memory. Time processes and their criterion durations will be referenced in memory, and subjective responses can be plotted against a range of task-related scalar expectations. Although the location of a hypothesized internal clock has not been determined, researchers have noted that neural oscillations and the firing of neural assemblies build a time system in the brain that might be responsible for time perception and production (see, e.g., Buhusi and Meck, 2005; Teki et al., 2012). Brain areas that seem to be involved in time processing include the insula (Wittmann et al., 2007b), the orbitofrontal cortex (Berlin et al., 2004), the auditory cortex (Sysoeva et al., 2006), the cerebellum (Teki et al., 2011), the posterior right parietal cortex (Alexander et al., 2005), the basal ganglia (Teki et al., 2011), and specific motor areas (Macar et al., 2006).

More specifically, dopamine, serotonin, and acetylcholine have been found to be involved in the processing of temporal information. Different systems appear to be involved in the processing of shorter and longer durations. The *dopaminergic* system apparently governs the processing of durations up to about 0.5 or 1 s, whereas the *serotonergic* system governs the processing of durations in the seconds-to-minutes range (e.g., Wittmann et al., 2007a,b; Wackermann et al., 2008; Sysoeva et al., 2010). Finally, the memory for temporal events and attention to it seem to be linked to the *acetylcholine* system in the frontal cortex. “These two systems are connected by frontal-striatal loops, thus allowing for the completion of the timing sequences involved in duration discrimination” (Meck, 1996, p. 227).

As we have discussed above, music may affect both the pacemaker, by influencing physiological arousal, and the memory and reference processes, by distracting attention. Clock speed (pacemaker) can be influenced by dopaminergic manipulations, the integration of longer durations can be influenced by serotonergic manipulations, and memory processes (reference) can be influenced by cholinergic manipulations (Meck, 1996, 2005). It remains a task for future research to determine in much more detail if and how music affects these neurotransmitter systems.

Because music can induce emotions it might also modulate the activity of the *locus coeruleus norepinephrine system*, which influences the speed of internal processes such as working memory performance, decision processes, or behavioral responses (see Arstila, 2012). As we have described above, Arstila has argued that the altered speed of inner processes is plotted against the memory of how much time things usually take, which results in the experience of a specific speed or flow of time.

Baer et al. (2013; see also Teki et al., 2012) argued that there might be a dissociation between event-based and emergent timing mechanisms. Event-based mechanisms (such as finger tapping) may rely on an inner pacemaker whereas emergent timing mechanisms (such as circle drawing) are embodied skills that do not require a pacemaker. The authors found that musicians are better than non-musicians only in event-based timing tasks but not in emergent timing tasks.

Another branch of neurobiological research has been concerned with music, emotions, absorption, and ASC phenomena. Menon and Levitin (2005) demonstrated a sequence of activation for music perception and emotion. They found that, starting from the auditory center, the cascade of activation initializes changes in parts of the frontal cortex and from there proceeds to the mesolimbic reward centers, finally reaching the nucleus accumbens, releasing waves of dopamine. As expected, the cerebellum and the basal ganglia—dopaminergic regions of the brain involved in motor and timing processes and the analysis of rhythm and meter in music—became active as well. Phasic increases in dopamine release happen when meaningful objects are in the focus of attention; the higher the personal meaning and valence of the object in focus, the more dopamine is released (Yacubian and Büchel, 2009).

The functional changes music elicits in the frontal brain regions have received special attention as part of Dietrich's (2003) concept of *hypofrontality*. The frontal and precentral lobes of the brain constitute the explicit system, the “active part” that organizes top-down processing and structures motor activity and output; the posterior lobes (postcentral, temporal, parietal, and occipital) are the “receiving parts”—the implicit system of the brain. They are primarily concerned with sensory input and with processing bottom-up information in the implicit system. Dietrich (2003) proposed that the prefrontal cortex, the highest integrating component in a hierarchy of cognitive functions, is deregulated in ASCs by ceasing to function in a “normal” way. This is the *hypofrontal* (reduced frontal brain activity) state, which can be compared to the state of flow, in which effortless information processing seems to take place. It enables the temporary suppression of the analytical and meta-conscious capacities of the explicit system while being relaxed and absorbed in the flood of sensory input, allowing people to see things from a different perspective or to arrive at new creative solutions to problems (Dietrich, 2004).

Note that what we have presented here is more a selection of prominent models than an exhaustive list. Neurophysiological models are extremely diverse; some of them overlap in their arguments to a certain extent and most of them exhibit a lack of empirical underpinning. There is as yet no agreement on these models, which makes research on the representation of time quite difficult.

### Discussion and suggestions for further research

Regarding the impact of music on the representation of time, we can draw the following conclusions: (1) Typically, music leads to an underestimation of the length of time intervals; (2) attention appears to be one of the most important factors when explaining the impact of music on time perception; and (3) when taking additional variables into account—such as arousal, emotional expression, familiarity, and likability—the results become inconsistent and are no longer satisfactorily explained by the existing approaches. As we have delineated above, attention might be the mediator between these additional variables and time perception. For instance, attention to the passage of time might decrease as a function of the pleasure evoked by the music because the listener is captivated or distracted. The same may apply to music-induced arousal and familiarity. Moreover, we simply do not know if these relationships—if they exist—are linear. Regarding arousal and familiarity, they might just as easily be U-shaped, which would make matters even more complicated.

As shown, many models of human time processing have been consulted in efforts to understand the effects of music on the representation of time. And as we have shown, each of these models can account for a certain proportion of the observed phenomena. However, there is no single model that can account for all phenomena comprehensively. Moreover, while some of the models can probably be integrated, others predict contradictory effects (see Fraisse, 1984). The models differ in four ways: they follow different approaches (psychological, phenomenological, neurophysiological); they focus on different phenomena (duration judgments vs. subjective time flow); they adopt different research paradigms (prospective vs. retrospective time estimation); and they have been used to explain different effects from different research settings (time estimation as a primary or a secondary task). Above, we discussed the first two issues. We now go into more detail regarding the latter two before presenting suggestions for further research.

#### Prospective vs. retrospective time estimation

Prospective time estimation occurs when respondents in a study are aware that they will have to estimate the length of intervals. Retrospective time estimation occurs when respondents have to estimate the length of an interval or their experienced flow of time only after the interval is over. Note that in empirical research, judgments about time intervals are almost always given retrospectively, that is, after the experience. However, in prospective settings, the judgment is built *during* the experience and reported afterward, whereas in retrospective settings, the judgment is both built and reported only *after* the experience. Apparently, completely different processes are involved when people are asked to estimate intervals either prospectively or retrospectively (see, e.g., Wittmann, 2009). The most prominent difference is the degree of attention directed toward the time itself. Prospective settings make people aware of the task of time perception and they will try to infer the elapsed time as best they can. In retrospective settings, people do not know that they will have to estimate time intervals and thus will not specifically focus their attention to the passage of time.

There is a dissociation of processes involved in the two types of time perception (see, e.g., Zakay and Block, 2004). Sitting in silence will cause the impression that time is dragging because there is just nothing going on that would demand cognitive processing, resulting in constant awareness of the elapsing time (prospective). However, when asked some time afterward, the person who has sat in silence would claim that not much time had elapsed, because there are simply no memories about that period of time. In retrospect, time shrinks. The same occurs when people are engaged in routine or boring activities—which likely accounts for the impression that time seems to go faster when people get older. In contrast, doing something interesting or new will cause the impression that time flies because attention is only occasionally directed toward the passage of time (prospective), leaving the impression that time has jumped from one point in time to another point minutes or hours later. In retrospect, however, there will be many memories about this period of time, which will lead to the conclusion that much more time has elapsed than actually did.

The challenging point is that attention-based models of time perception are models that account for prospective time perception only. Specifically, the attentional gate model involves the opening and closing of an “attentional gate”, which apparently depends on the specific awareness that an estimation of time intervals is going on. These models can therefore not account for the dissociation of prospective and retrospective timing. Memory-based models, on the other side, are more capable of explaining retrospective timing. They cannot explain the phenomenon of subjective time flow, however. All the remaining models (phenomenological models, absorption/ASC models, neurophysiological models) can, in principle, account for both prospective and retrospective timing, although not all of them explicitly refer to the difference between the two types of timing.

#### Time estimation as primary vs. secondary task

A second methodological issue that might account for inconsistent empirical results is whether the time estimation task is a primary or a secondary task. Because attention turns out to be a crucial component in time perception it makes a difference how it is allocated to different tasks that are present at the same time. In most of the studies mentioned above, the perception of time intervals was the only task participants had to deal with. In these types of studies it is easy to investigate the influence of music and even to compare prospective and retrospective timing. However, there are also studies where participants had to deal with time perception as a secondary task. These types of settings make it hard to investigate how people's attention is distributed to the primary and the secondary task. Subsequently, it is hard to know whether prospective timing occurs (when enough attentional resources are left) or not (when the primary task does not leave any attentional resources for time processing). The overestimation of elapsed time under the influence of preferred music—as compared to experimenter-selected music—found by Cassidy and MacDonald (2010) is a pertinent example. Participants had to work on a driving task on a computer while prospective time estimation was their secondary task. The authors did not report measures of the allocation of attentional resources. It is conceivable that there were no attentional resources left so that prospective timing did not actually occur. If this was the case, judgments would have been made in retrospect only, leading to the phenomenon just described: a joyful experience—a result of listening to preferred music—would afterward seem to have lasted longer. This might be an explanation of the unexpected finding in this study. Note, however, that this is only one possible explanation—there might be further effects occurring with time perception in dual-task situations that are severely under-researched.

#### Suggestions for further research

Based on the observation of inconsistent empirical results and inconsistent explanations, we would like to delineate some suggestions for future research on music and time. (1) Experimental works should more consistently state whether they adopted a prospective or a retrospective approach and what exactly has been measured. (2) Research conducted using either of the two types of timing should address additional variables that potentially influence the impact of music on time estimation: arousal, emotional expression, likability, familiarity, absorption, and the current goals and concerns of the listener. (3) Certainly more theorizing is needed not only to integrate these variables into the existing models of time perception, but also to integrate the two types of timing into a comprehensive model. This would also mean rethinking the role of attention as a potential mediator for all effects of specific variables on time perception. Developing more sophisticated structural models is recommended. (4) More theorizing is needed regarding the differences that might arise when timing is done as either a primary or a secondary task. Specifically, as one of the first steps, it should be analyzed how attention is distributed across different tasks at the same time. (5) Finally, the question of how prospective timing actually occurs is not well-understood and needs a theoretical and empirical boost. What exactly do people do when judging the flow of time at a particular point in time? What actually do they “perceive” in order to make these judgments? The experience of a particular point in time—the subjectively experienced now or present—appears to have an impact on the judgment of longer durations, which is still under-researched, however (see Pöppel, 2004; Wittmann, 2011; see Le Poidevin, 2009, for a theoretical discussion). We suggest using continuous online measurements (such as continuous sliders) as well as more qualitative work (e.g., grounded theory) to figure out what, exactly, is going on. Furthermore, we have delineated phenomenological approaches to the subjective experience of time. These models provide a reasonable explanation for the experienced speed or flow of time. Together with memory-based models they might be able to explain the whole range of prospective and retrospective timing. However, we need many more empirical studies to buttress this notion.

There are further suggestions for further research regarding the *subjective experience* of time under the influence of music. The most obvious question is how the experimental evidence relates to the subjective experiences. Subjective experiences are known from research about intense, outstanding, trance-induced, ecstasy-induced, or drug-induced experiences with music. That is, all these experiences certainly lie at one extreme end of a music experience scale. The music listening experiences that have usually been investigated—or created—in experimental studies are certainly not that extreme. We must therefore assume that the experiences differ significantly between these two types of settings and that we are thus not yet able to compare the experimental evidence and the evidence from subjective reports directly. However, as just indicated, we believe that all these subjective experiences lie on the same scale and differ only quantitatively (see also Schäfer et al., 2013). We could call the scale “music experience” (although the concept of *musikerleben* once proposed by Behne (1997), is maybe a better descriptor).

Apparently, although it is possible to elicit frisson experiences in a lab setting to some extent (e.g., Blood and Zatorre, 2001), it is not that easy to bring intense/outstanding experiences with music into the lab. But it should be possible to do it the other way round and gather empirical data about time interval estimation in the field. Doing so could help improve our knowledge about the relationship between music and the representation of time in several respects: (1) It could be analyzed if a scale of music experience or musikerleben—covering usual music experience as well as intense experiences with music—can be developed. If it cannot be developed we would know that music experiences are categorical. This is exactly the question that has been left open by Flaherty's (1999) model: Do experiences with music that leave traces in memory differ quantitatively or qualitatively from experiences that do not leave traces in memory? (2) Either way, this knowledge would enable us to integrate the evidence from subjective reports and the empirical evidence on prospective timing. (3) Further theorizing and empirical research should then occur regarding the integration of different models of psychological timing. As delineated above, Flaherty's (1999) model appears to explain the subjective experience of time quite well. We deem it absolutely promising to combine this model with attention-based and memory-based models of prospective and retrospective timing. Such an integrative approach would have two additional advantages. First, in the course of investigating attention-based and memory-based models of time perception, knowledge has accumulated about its physiological and neuronal correlates. This knowledge would enrich an integrative model and render it more comprehensive. Second, Flaherty's (1999) model proposes that personal, social, motivational, and emotional variables would mediate the way music influences the experience of time. As discussed, these variables have not yet been recognized sufficiently and have led to inconsistent empirical results, respectively. They should be incorporated as well.

## Music and the representation of space

Whereas altered representation of time has been investigated to a greater or lesser extent in empirical studies, there are far fewer investigations on the representation of space while listening to music. By “representation of space” we mean the way people perceive the space around them, such as the distance, size, shape, and movement of objects and other persons. Note that we do not present research on the effect of music on spatial abilities (cf., the Mozart effect) because it is still unclear if this effect really does incorporate a change in the representation of spatial objects (e.g., Husain et al., 2002). There is more anecdotal evidence than assured knowledge of how the representation of space is altered when people are captivated by music.

### Empirical evidence of the influence of music on the representation of space

In their descriptive system on strong experiences related to music, Gabrielsson and Lindström Wik (2003, p. 174) found one broad category—*changed experience of situation, body and mind, time and space, wholeness*. This category seems to contain far more descriptions of changed experience of time than of changed experience of space. When the respondents referred to space, they usually reported that it ceased to exist.

Regarding changes to the usual representation of space, Herbert (2011) found people to have a sense of music blending with the environment, to use music to “give a filmic quality to their surroundings” (p. 59), and to experience their surroundings as “choreographed.” Typically, the visual sense seems to be enhanced but awareness is focused only on select objects of the external environment. The more the person is captivated or absorbed by the music the more experience is characterized by “narrowed awareness, inwardly focused attention, and a lessened orientation to reality” (p. 190).

There have been some studies showing that the representation of spatial magnitudes can be associated with musical characteristics. For instance, it appeared that high frequencies convey a better representation of space than low frequencies because the former are easier to localize (Fachner, 2000; see also DeSouza et al., 1974). The perception of space under the influence of music may also be influenced by *motion imagery* induced by certain musical elements. When investigating the relationship of specific musical and motional parameters, Eitan and Granot (2006) found that aspects of timing were related to speed, aspects of pitch contour were related to vertical and horizontal movements (pitch fall moves downward and to the left) and to distance (pitch fall is associated with decreasing distance), and aspects of loudness were related to distance and energy. Christensen (1996) argued that intensity, timbre, and (pitch) space of auditory events are vital aspects of microtemporal localization strategies within the physical environment; although music may represent nomothetic “real-world” aspects of spatial relations [“high” vs. “low” (pitch)], it actually creates a virtual time-space within listeners based on ideographic metrics and states. Dolscheid et al. (2013) concluded that metaphors such as high vs. low pitch—which is thin vs. thick pitch, light vs. heavy pitch, young vs. old pitch, or weak vs. strong pitch in different languages—must all be partly innate but are reshaped and strengthened by language. Musical metaphors facilitate spatial associations and can therefore have an impact on the subjective experience of the spatial environment.

In general, rhythm appears to be the predominant musical feature inducing ASCs (see Fachner, 2006). Baldemair (2003) suggested that techno dancers lose their ordinary sense of space when they experience states of vertigo owing to the music's characteristics (fast tempi, monotonous sounds, unexpected breaks). She also suggested a need for temporal decomposition of self-awareness that can be managed through music and dance. Hill (2002, as cited in Becker-Blease, 2004, p. 92) described new-age and ambient music as “celestial or cosmic music [that] removes listeners from their ordinary acoustical surroundings by creating stereo sound images of vast, apparently dimensionless spatial environments, in a word—spacey.” When listeners described their experiences under the influence of mind-altering drugs, subjective alterations of “acoustic space” were mentioned (Tart, 1971).

There is also evidence that musicians often experience music as a spatial–temporal image. When investigating predictors of music sight-reading ability in high school wind players, Gromko (2004) found that “reading music is a spatial process that may be like the reading of two-dimensional architectural drawings that are comprehended as three-dimensional objects. In other words, when skilled musicians read musical notation, they may mentally represent the sound as an image with spatial and temporal dimensions” (p. 12). When investigating novice musicians, McLachlan et al. (2010) (p. 12) found that “sound attributes such as pitch, loudness, duration, and location are spatially encoded in a multidimensional array in ASTM [auditory short term memory].”

In sum, it has been found that music can (1) distort the ordinary perception of space, (2) cause the experience of space ceasing to exist, (3) be used as a source of information about the movement of objects through space, and (4) trigger spatial mental images. Apparently, most of these experiences again have been reported in very intense or outstanding listening situations. To date, there are no studies that investigated if there are any changes in the representation of space under the influence of music in usual everyday listening situations.

### Explanations for the influence of music on the representation of space

Due to the scant evidence regarding this issue it is not possible to provide a comprehensive explanation. Nonetheless, we would like to delineate some approaches that can help explain the effects of music on the representation of space. Blood and Zatorre (2001) demonstrated that the visual cortex shows decreased activity during chill experiences induced by music. This may be an explanation of the illusion that space ceases to exist. This line of neurobiological research has to be linked with subjective experiences in much more detail in the future. In addition, more research is needed to clarify how the processing of time and the processing of space are intertwined. Changes in time processing might cause changes in the representation of space, and vice versa (see final discussion).

An inquiry on out-of-body experiences has shown that people are more likely to experience an ASC in immobility, when lying supine or sitting (Zingrone et al., 2010), when the focus of attention can turn inward, and more afferent information is processed—a finding that corroborates Rouget's (1985) concept of ecstasy. Deep experiences of hypnosis in immobility have been found to be accompanied by alterations of body image (Cardena, 2005). It seems that the moment of an ASC experience is related to a motionless, inwardly turned attention, evoking images and emotions, while the person is perceiving a temporary disappearance of spatial relations and perspective on his or her own body in a private space.

### Discussion and suggestions for further research

Apparently, we are not able to provide a final conclusion about the influence of music on the representation of space. We have, however, learned that music can have such an influence and that it has typically been observed during intense or outstanding musical experiences. Most of the reported experiences refer to a distortion of spatial representations or the feeling that space simply disappears. Regarding approaches to explaining these effects, there is not much on the table either. We can summarize that there are two promising approaches so far. One involves the neurobiological finding that changes in the activation of the occipital cortex due to music listening might change the way listeners process and experience spatial information. The other approach would be one that highlights the role of attention. The majority of subjective reports about an altered representation of space during intense experiences with music have emphasized that attention has turned inward and that the representation of the outer world has faded into the background. We have argued above that space and time are represented rather than perceived. That is, they are mental constructions requiring mental processing, which is influenced by the attention that is directed toward it. A lack of attention might result in distorted or inaccurate mental images that differ from the usual images we have about the outside world. We believe that attention plays a central role in the processing of spatial information.

Thus, there is a long list of questions for future research. (1) What role does attention play in the representation of space? (2) How are the representation of space and time intertwined? Could attention be the central variable that accounts for effects of music in both areas? (3) Can neurobiological effects of musical experiences be related more concretely to subjective experiences? (4) Can distortions in the representation of space be found in experimental settings? This question bears a large potential for empirical studies. Potential effects of music on the processing of spatial information could be analyzed using eye-tracking or spatial perception tasks. (5) Finally—as we mentioned regarding the representation of time, as well—we want to know if music always affects spatial processing to a certain degree, and not only during intense experience. Again, this degree of influence could be related to the degree of what we called “music experience” or “musikerleben.”

## Final discussion

Music affects not only our emotions, motivation, and actions but also the way we experience the world on a very basic level: the experience of space and time. Music can make time go by slower or faster and it can even create the experience that time disappears. It can lead to the impression that a period of time has had a shorter or longer duration than it actually had. It can also make objects in the world or the world itself appear to look different or to even disappear. We have learned that there is considerable research about the influence of music on the experience of time, in terms of both time interval estimates and subjective experiences, but that there is a considerable lack of research on the influence of music on the experience of spatial information. We have also learned that there have been attempts to explain all these effects, although, again, there have been more—and more sophisticated—approaches regarding time than regarding space. We have delineated several suggestions for further research, the most important of which deal with the clarification of the role of attention, the integration of experimental and subjective evidence in models of psychological timing, the integration of knowledge regarding the observed effect of music on the representation of both space and time, and the investigation of a variable we called music experience.

Space and time are the two concepts with which we describe our physical existence in the universe. There is, however, a dissociation between these two concepts: We can perceive space more or less directly but we cannot perceive time (DeLong, 1981). Of course, our mental images of space or objects in space are also a representation and are affected by learning and expectation, among other influences, but there is a physical basis that we can perceive through our senses. Time, in contrast, does not have a comparable physical basis; we are not able to perceive time through our senses. This is evidenced by the way we think about time, namely, through spatial metaphors. One classic example is that people in the Western world usually associate the past with the left space and the future with the right space. Bonato et al. (2012, p. 2270) recently reviewed the relevant literature and concluded that “overall, there should be no doubt that the processing of time is not independent of the processing of space.” They also discussed some approaches to explaining this space–time interaction. For instance, the *mental timeline model* holds that temporal information is represented by a spatial timeline. Hence, temporal processing would be affected by spatial information but not vice versa. Some studies have found that this is the case (see Casasanto and Boroditsky, 2008; Calabria et al., 2011). This dissociation probably is the reason for our finding that space is not influenced by music listening as much as time is. Spatial information seems to be much less malleable and prone to representational distortions than is temporal information.

We now summarize what we have learned regarding the role of embodiment in the impact of music on the representation of space and time and make some final remarks.

### Music, space, time, and the embodied mind

Is the experience of music an embodied experience? Based on our review, the answer is clearly yes. The overarching assumption of the embodied mind idea is that the shape of the body influences the shape of the mind. The body is a physical object that is positioned in and can move through physical space and time. The body's positioning and movements are perceived by the sensory system and thus provide the basis for the mental representation of space and time, which is intrinsic to all cognitions and behaviors. We have shown that music has the power to transform the representation of spatial and temporal magnitudes, which results in experiences regarding the speed of time, the duration of time periods, and the appearance of space that are detached to a varying degree from their physical basis. Music is shaped time, it unfolds in time, and it is made through certain actions with real things (instruments or vocal folds and facial muscles). The experiences music elicits—including their spatial and temporal aspects—therefore mirror the enactive character of music, where “enactive in this context signifies that perception (and experience more generally) is characterized by a structural coupling between the agentive body and its environment in a way that generates action-oriented meaning” (Gallagher, 2013, p. 141).

**Figure 1:**
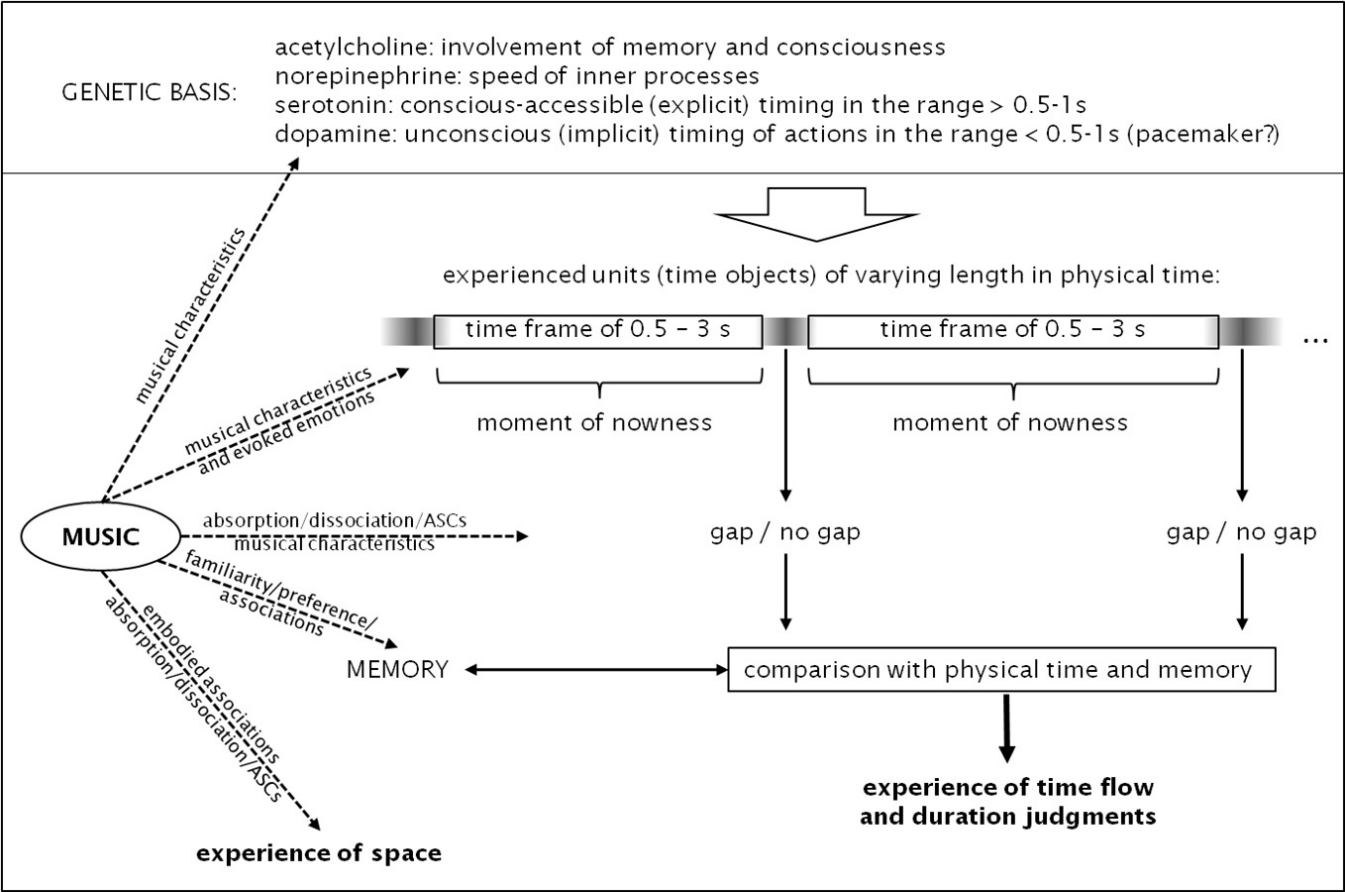
**Summary of the numerous influences of music listening on the representation of space and time.** See text for a detailed explanation. ASCs, altered states of consciousness.

As we noted earlier, there are a large number of psychological and neurophysiological theories/models of time perception and space perception that can all contribute to explaining the effects of music. We have also noted that these models argue on different levels (e.g., objective measurements of duration judgments vs. subjective experiences) and are hard to reconcile. Nevertheless—or precisely because of this—we would like to risk an attempt to provide a working model of the influences of music on the representation of space and time, which includes all the empirical results and theoretical notions that fit together well (see Figure 1). There are genetic factors that—mediated by dopamine, serotonin, norepinephrine, and acetylcholine—govern the processing of time intervals, the speed of internal processes, and the incorporation of memory and self-related concepts. There is a dissociation between shorter intervals (up to 0.5 or 1 s), apparently responsible for the unconscious or implicit timing of behavior, and longer intervals that provide the time frames for consciousness-accessible units (time objects). These time frames can range from 0.5 to 3 s of physical time and are separated by the desynchronization/reorganization of cell assemblies in the brain (gaps). Each of these units is experienced as a moment of nowness or as the present. Gaps facilitate the conscious comparison of a certain number of units with both physical time (that can be read from a watch or inferred from circadian rhythms) and memory (how long the current experience usually lasts). This comparison leads to the experienced flow of time and provides the basis for judgments about given durations. Both subjective time flow and “objective” duration judgments are malleable because the length of the units is variable, just as is the occurrence and frequency of the gaps. These two parameters are influenced by a large number of factors, including the present situation, current environmental cues or stimuli, other ongoing activities, the physiological condition of the body, and the person's current mood, affective disposition, concerns and motivations, age, and personality.

Music, as an embodied or enactive experience, can affect the processes described in the model in several ways. Musical characteristics can influence the length of the units and the occurrence and frequency of the gaps. The number of notes or chords that are tied together to a single phrase or unit is variable and there might be fewer or even no gaps between the units when musical phrases span a larger number of notes or chords. When music leads to captivation, absorption, dissociation, or other kinds of ASCs this can influence the occurrence and frequency of gaps. Fewer gaps will occur the more the listener is absorbed by the music. Whenever there is a gap, however, the current experience can be compared with memory, resulting in a relative feeling of time flow that also provides the basis for duration judgments (both prospectively and retrospectively). Memory, of course, is also affected by musical experiences in the past in terms of familiarity, preference, and associations.

Regarding the representation of space, we have concluded that there are practically no reports about alterations in the perception of real objects caused by music listening. What we have are some embodied associations between musical characteristics and real-world objects as to their size or energy and their movement through space. For instance, faster music is associated with more energy than slower music; louder music is associated with more energy than quieter music; lower pitch is associated with the resonance of larger objects while higher pitch is associated with smaller objects (see, e.g., Huron, 2006). Such associations may also be responsible for music imagery. We found more evidence regarding the experience that the outer space ceases to exist under the influence of music—at least in situations of high absorption. The reason for this effect might be quite simple. Given that music listening is an embodied experience, it is associated with specific actions or movements in space. These associations might interfere with the perception of the outer world that is currently present. This is why the focus of awareness shifts inward to facilitate unfolding of the embodied associations and emotions. It might also explain why we like to close our eyes when we are (or want to get) captivated by the music we listen to.

We consider this model more a collection of consistent evidence and theoretical approaches than a conclusive model of causal processes and relationships. Many of the proposed relationships are speculative and should be investigated in future research.

### Final remarks

We believe that understanding the effects of music on the processing of time and space can lead to insights on the emergence of music in human evolution. We have mentioned that rhythm is a central aspect of music and that rhythmic entrainment might have played an important role in the coordination of common activities in prehistoric times (see also Huron, 2006). Interest in the role of sounds and music in the course of human evolution has started to grow again (see, e.g., Horowitz, 2012) and it has been argued that much more research is needed in this respect:

The role and effect of sound and the human experience of sound in archaeological environments is severely under-researched. Sound is a primary source of information about the world, and the human experience of sound shapes many of the ways in which we interact with the world and with each other (Cross and Watson, 2006, p. 107).

Brandt et al. (2012, p. 13) have gone one step further to conclude that musicality is essential to language acquisition: “Listening to music may give us insights into how language sounds to us before we understand it—and how we experience our world before we have words.”

Our results may also be deemed inducement to rethink the concepts of invariant, physical space and time in psychological research. Obviously, the suggestions we have raised concerning future research on approaches and models explaining the effects of music on the experience of space and time are of a more far-reaching nature. In particular, our suggestions regarding the models of time perception are quite general and not restricted to the influence of music. We believe that the biggest task in the future will be to reconcile the heterogeneous models of time processing, thereby integrating the psychological, the phenomenological, and the neurophysiological levels (see also Michon, 1993, who proposed a framework of seven criteria that should be taken into account when formulating a coherent theory of psychological timing).

Not least, we would applaud any attempt by psychologists to pay more attention to the subjectivity and dynamics of spatial and temporal experiences. This has been done rather reluctantly in the past although this is not a new postulation: “The future of cognitive science may thus lie in a delicately negotiated union between the familiar framework of computational, representational and information-processing description, and the challenging and temporally charged project of dynamical analysis” (Clark, 1998, p. 370). Yet, the ancient Greeks distinguished *chronological* time—conventional time by the clock oriented to the geophysical concept of time—from *kairological* time—personal, individual time. Kairological time emerges from personal perception of time and periods of time. The right time for doing something, deciding or acting in the “here and now” (Aldridge, 1996) are kairological expressions of time. Anticipation of what is coming up next and what is needed to be perceived is surely of vital interest to humans so that it is important to know not only “where to place attention, but also when” (Eagleman et al., 2005). Pleasant situations fly by whereas unpleasant ones seem to last for hours. People experience special moments or situations that interfere with their personal set of emotions, habits, and beliefs. Depending on the situation, time is perceived as decelerated or accelerated. Thus, the concept of kairological, personal time is much closer to what people really experience than is the concept of a constant physical, chronological time. We are well on the way to bringing this ancient knowledge back into psychological research.

### Conflict of interest statement

The authors declare that the research was conducted in the absence of any commercial or financial relationships that could be construed as a potential conflict of interest.
